# WRB and CAML Are Necessary and Sufficient to Mediate Tail-Anchored Protein Targeting to the ER Membrane

**DOI:** 10.1371/journal.pone.0085033

**Published:** 2014-01-02

**Authors:** Fabio Vilardi, Milena Stephan, Anne Clancy, Andreas Janshoff, Blanche Schwappach

**Affiliations:** 1 Institute of Molecular Biology, University of Göttingen, Göttingen, Germany; 2 Institute of Physical Chemistry, University of Göttingen, Göttingen, Germany; Ecole Polytechnique Federale de Lausanne, Switzerland

## Abstract

Tail-Anchored (TA) proteins are inserted into the endoplasmic reticulum (ER) membrane of yeast cells via the posttranslational Guided Entry of Tail-Anchored protein (GET) pathway. The key component of this targeting machinery is the ATPase Get3 that docks to the ER membrane by interacting with a receptor complex formed by the proteins Get1 and Get2. A conserved pathway is present in higher eukaryotes and is mediated by TRC40, homolog of Get3, and the recently identified membrane receptors WRB and CAML. Here, we used yeast lacking the *GET1* and *GET2* genes and substituted them with WRB and CAML. This rescued the growth phenotypes of the GET receptor mutant. We demonstrate that WRB and CAML efficiently recruit Get3 to the ER membrane and promote the targeting of the TA proteins *in vivo*. Our results show that the membrane spanning segments of CAML are essential to create a functional receptor with WRB and to ensure TA protein membrane insertion. Finally, we determined the binding parameters of TRC40 to the WRB/CAML receptor. We conclude that together, WRB and CAML are not only necessary but also sufficient to create a functional membrane receptor complex for TRC40. The yeast complementation assay can be used to further dissect the structure-function relationship of the WRB/CAML heteromultimer in the absence of endogenous receptor proteins.

## Introduction

Tail-Anchored (TA) proteins are membrane proteins characterized by a single transmembrane domain within the last 40 to 50 C-terminal residues, the absence of a cleavable signal sequence and the orientation of the N-terminal functional domains towards the cytosol [1]. Their unique topological features exclude TA proteins from Signal Recognition Particle (SRP) -dependent cotranslational targeting to the endoplasmic reticulum (ER) membrane. In fact, TA proteins are released from cytosolic ribosomes only after the translation has been completed and the newly synthesized polypeptide is targeted to the destination membrane posttranslationally [2]. As TA proteins carry a hydrophobic transmembrane segment released from the ribosome into the aqueous cytosolic environment, cells have evolved a chaperoning system to shield this aggregation-prone region of the protein and mediate its membrane targeting. The homologous proteins, yeast Get3 (Guided entry of TA proteins 3) and mammalian TRC40 (Transmembrane domain Recognition Complex 40 kDa subunit) were identified as soluble targeting factors involved in the incorporation of TA proteins into the ER membrane [3]–[5].

Targeting specificity to the right membrane is ensured by the presence of receptor proteins at the ER. In yeast two integral ER membrane proteins named Get1 and Get2 create a receptor complex that binds the Get3 dimer and allows the insertion of TA proteins [3], [6]. Structural studies have revealed that the cytosolic domain of Get2 binds a negatively charged surface in the Get3 dimer and works as tethering site for Get3 prior to its more stable binding to the coiled coil domain of Get1. This domain induces the release of the substrate TA protein and of ADP molecules from the nucleotide binding sites of the Get3 ATPase. Get1 and Get2 were reconstituted in proteoliposomes to demonstrate that they are necessary and sufficient to mediate membrane integration of TA proteins by Get3 [7], [8]. However, it remains unclear if and how the GET receptor comprised of Get1 and Get2 contributes to the actual integration of the TA protein transmembrane segment into the lipid bilayer. In fact, there is currently no insight into the structural or functional properties of the transmembrane region of the GET receptor.

In higher eukaryotes, the molecular nature of TRC40 receptors has only recently been clarified. Based on moderate sequence homology, WRB was suggested as putative Get1 homolog [1], [3] and later characterized as an ER resident membrane protein able to bind TRC40 [9]. A mammalian Get2 homolog could not be similarly identified based on sequence conservation. A recent study used TRC40 to affinity-purify mammalian receptor proteins from brain membrane preparations and demonstrated co-purification of the calcium-signal modulating cyclophilin ligand (CAML) with WRB. An active role of CAML in TA protein membrane insertion was demonstrated using an RNA-silencing approach [10].

CAML was originally identified for its ability to bind cyclophilin B and was initially linked to calcium mobilization [11]. It is known that CAML plays an active role in development and survival of peripheral follicular B cells [12] and T cells [13]. Moreover, CAML interacts with TACI, a cell surface receptor in T lymphocytes, to regulate signaling and recycling of epidermal growth factor (EGF) receptor [14] and binds gamma-amino butyric acid (GABA)_A_ receptors to mediate their membrane trafficking [15]. Deletion of CAML is lethal at early embryonic developmental stages [14] while its conditional knock out in the inner ear cells causes deafness in mice [16].

A general role in TA protein biogenesis could well explain the diverse phenotypes linked to the manipulation of CAML *in vivo* and its impact on several signaling and trafficking pathways. However, it has not yet been shown that WRB and CAML are sufficient to form a functional GET receptor. Hence, we sought to further corroborate the conclusion that CAML is indeed the elusive functional Get2 equivalent in the mammalian GET receptor. To this end we used a complementation approach in yeast and found that together, WRB and CAML can functionally replace Get1 and Get2 in yeast cells. The WRB/CAML complex creates a functional receptor complex that recruits Get3 to the ER membrane and ensures targeting of TA proteins *in vivo*. We also provide binding parameters that characterize the interaction of TRC40 with the receptor complex at the level of the interacting cytosolic protein domains as well as in the context of the native mammalian microsomal membrane. Our data suggest that WRB and CAML are not only necessary but also sufficient to mediate TA protein insertion into the ER membrane.

## Results and Discussion

Recent studies have identified WRB and CAML as components of the membrane receptor complex for TRC40-mediated TA protein membrane insertion in higher eukaryotes [9], [10]. In order to study the molecular function of WRB and CAML in a heterologous system completely lacking either protein and putative additional mammalian receptor components, we expressed them in yeast cells devoid of the genes encoding Get1 and Get2 and confirmed their interaction by a split-ubiquitin based yeast two-hybrid assay (Figure S1A). Interestingly, we observed a substantial stabilization of WRB and CAML when the respective mammalian interaction partner was co-expressed (Figure S1B and C, lane 4). In contrast, no or only a minor effect on steady-state levels was observed when WRB and CAML were co-expressed with Get2 and Get1, respectively (Figure S1B and C, lane 3). Stabilization of the partner subunits in the context of the multimeric receptor has been previously described for Get1 and Get2 [3]. These data suggest that – as for Get1 and Get2– cells tightly regulate the steady-state levels of WRB and CAML, presumably via the degradation of proteins not incorporated into receptor complexes. We conclude that WRB and CAML form a complex when expressed in yeast.

**Figure 1:**
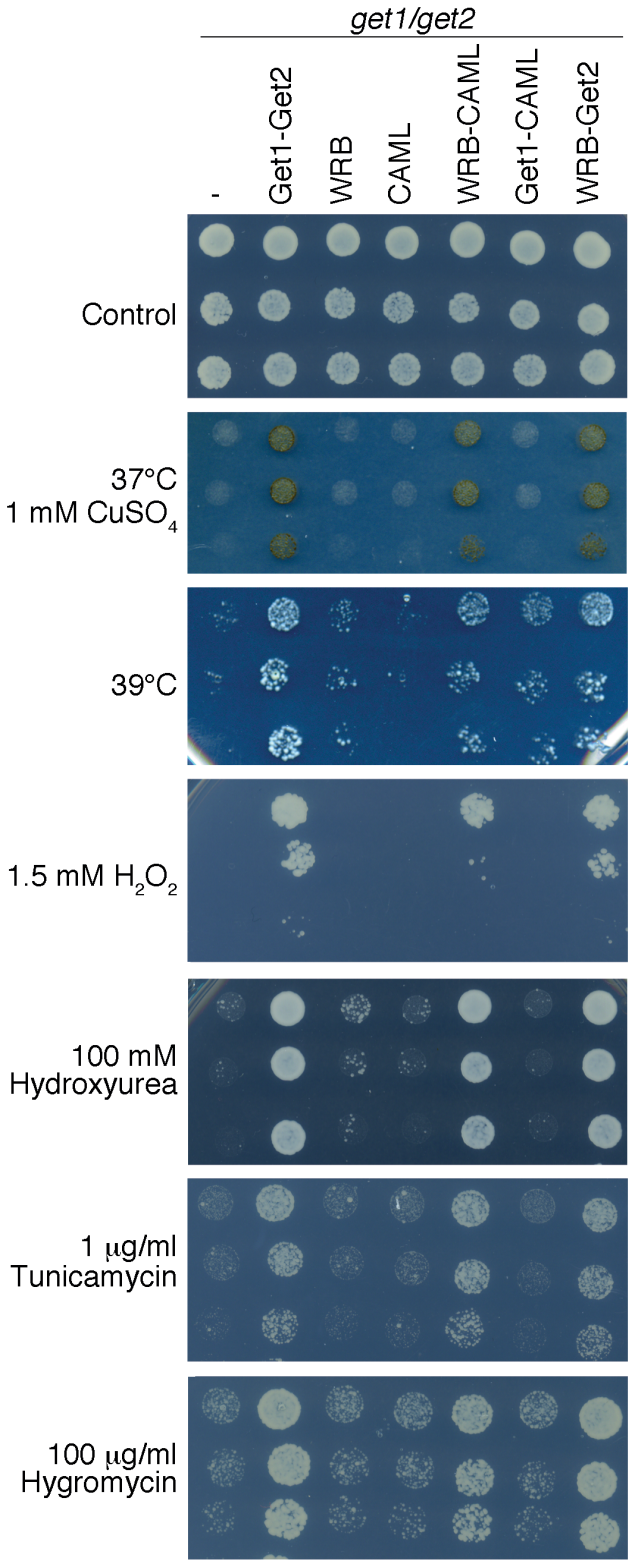
WRB and CAML rescue the growth phenotypes of *get1/get2* yeast cells. *get1/get2* yeast cells were transformed with combinations of WRB, CAML, Get1 and Get2 encoding constructs and serial dilutions spotted on different conditions: HC plates incubated at 30°C (control), 37°C+CuSO_4_, 39°C, H_2_O_2_, hydroxyurea, tunicamycin, hygromycin.

It was shown that yeast cells lacking Get1 and Get2 have severe growth defects under various conditions that cause oxidative stress or impair protein folding [3]. To investigate whether WRB and CAML can functionally replace Get1 and Get2 we transformed *get1/get2* cells with WRB and CAML either alone or in combination with each other. Cells expressing both WRB and CAML, but not cells expressing only one receptor component, could grow and cope with several stress conditions, rescuing all phenotypes observed for the mutant cells (Figure 1). Remarkably, WRB in combination with Get2 also rescued the growth defects of the GET receptor deletion strain. Excluding rescue at high temperature, no complementation was observed with coexpression of CAML and Get1. This suggests that the two proteins do not interact productively. It is unclear whether all phenotypes of the *get1/get2* deletion strain relate to TA protein targeting and what level of GET pathway activity is required to rescue them. Hence we include additional assays such as monitoring Get3-GFP recruitment to the ER membrane and the targeting of well-characterized TA protein substrates.

**Figure 2:**
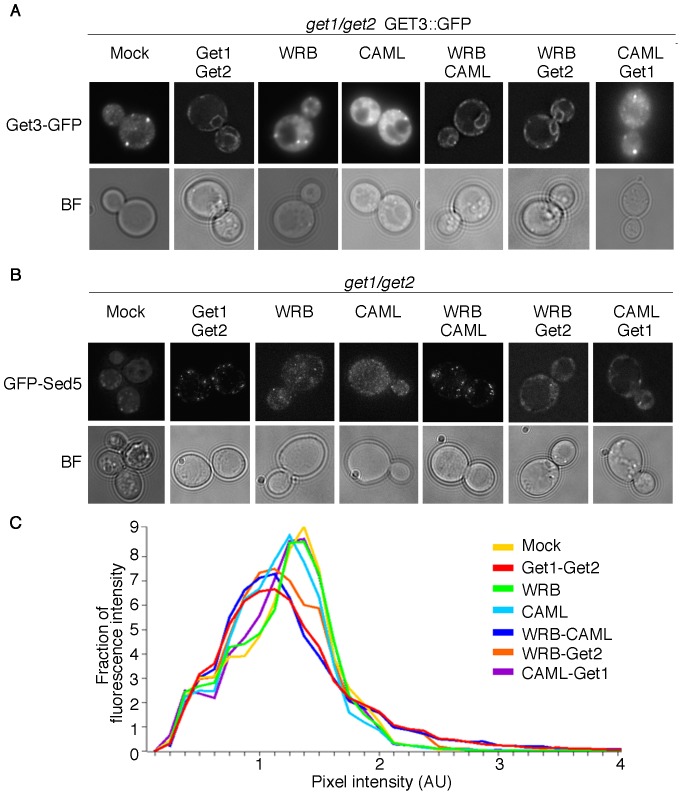
In combination, WRB and CAML rescue Get3 localization at the ER membrane and TA protein targeting. (A) *get1/get2* yeast cells carrying a genomically GFP-tagged version of Get3 were transformed with combinations of WRB, CAML, Get1 and Get2 encoding constructs. Subcellular Get3-GFP localization was analyzed by fluorescence microscopy. (B) *get1/get2* yeast cells were transformed with a plasmid containing the coding sequence of GFP-tagged Sed5 and combinations of WRB, CAML, Get1 and Get2 encoding constructs. Subcellular GFP-Sed5 localization was analyzed by fluorescence microscopy. (C) Images taken in (B) were quantified to determine the distribution of fluorescence across bins of different pixel intensity for each strain. A minimum of 41 cells was analyzed per strain.

Get3 in yeast and TRC40 in mammalian cells are present in two distinct sub-populations in the cytosol and at the ER membrane [3], [9]. Depletion of the receptors leads to loss of Get3 localization at the ER membrane, accumulation in the cytosol and in punctate cytosolic structures, where TA proteins are thought to aggregate in the absence of membrane targeting [3]. In yeast cells lacking *GET1* and *GET2* genes, a genomically GFP-tagged version of Get3 was detected in punctate cytosolic structures by fluorescence microscopy (Figure 2A) as previously observed [3]. Get3 ER localization was rescued when Get1 and Get2 or WRB in combination with CAML were reintroduced into the cells (Figure 2A). The data demonstrate that WRB and CAML can together, but not singularly, provide a docking site for Get3 at the ER membrane. In line with the functional effects observed for this combination (Figure 1), WRB was able to recruit Get3 to the ER membrane when coexpressed with Get2, whereas CAML and Get1 did not rescue Get3 ER localization. These results clearly show the interaction of Get3 with the heterologous WRB/CAML receptor and are in agreement with localization of Get3-GFP in single *get* mutants: ER association of Get3 was restored in *get1* GET3::GFP cells by transformation with a construct containing the coding sequence of Get1 or WRB (Figure S2A). On the other hand, Get3 targeting to the ER in *get2* GET3::GFP was rescued exclusively by Get2 but not by CAML (Figure S2B).

TA proteins that rely on the GET pathway to reach their destination membrane, accumulate in the cytosol and in deposition sites for aggregated proteins that contain Get3 and other chaperones when the pathway is impaired due to loss of the receptors or when energy becomes limiting [17], [18]. Distribution of the N-terminally GFP-tagged TA protein Sed5, a model substrate employed by several previous studies [3], [17]–[20], was monitored by fluorescence microscopy in *get1/get2* cells. The protein showed a diffuse cytosolic distribution with punctate structures resembling the previously described deposition sites for aggregated proteins (Figure 2B). Upon expression of Get1 and Get2, GFP-Sed5 localized exclusively to small, intensely fluorescent punctate structures previously shown to reflect Golgi membranes [3] and the background cytosolic signal disappeared completely. Individually, WRB and CAML were unable to mediate GFP-Sed5 targeting to the Golgi membrane, but when co-expressed they formed a fully functional receptor and proper Sed5 localization was restored (Figure 2B). Quantitative analysis of pixel fluorescence intensity [17], [21] demonstrates the concentration of fluorescent signal in intensely fluorescent punctate structures, representing Golgi structures, when a functional Get1/Get2 or WRB/CAML receptor is formed (Figure 2C). Briefly, functional complementation results in the shift of the peak of the distribution to the left (most pixels are of lower intensity because there is little cytosolic background) and in a shoulder reflecting a higher abundance of very bright pixels (found in the punctate Golgi structures). Fluorescence quantification reveals a partial rescue of TA protein insertion upon coexpression of WRB with Get2. This supports the notion that even a decreased level of proper TA targeting may be sufficient to sustain growth of cells expressing WRB and Get2 under stress conditions as shown in Figure 1. Rescue of TA protein insertion is not substrate-specific as WRB and CAML could ensure targeting of the ER resident TA protein Sbh2 (Figure S3), another substrate previously used to study the yeast Get pathway [3]. Taken together, these data strongly indicate that WRB and CAML are essential components of the receptor complex for TA protein membrane insertion. Importantly, our data strongly suggest that yeast Get3 can mediate TA protein insertion via interactions with the mammalian receptor proteins, despite the low level of sequence conservation between WRB and Get1 and despite the absence of obvious homology between CAML and Get2. This indicates a high level of functional conservation between the yeast GET and the mammalian TRC40 pathway. Moreover, these results confirm the previous *in vitro* observation that Get3 from the thermophilic fungus *Chaetomium thermophilum* can mediate the insertion of TA protein into canine ER-derived rough microsomes [22]. We conclude that when coexpressed, WRB and CAML functionally complement a GET-receptor deficient yeast strain.

**Figure 3:**
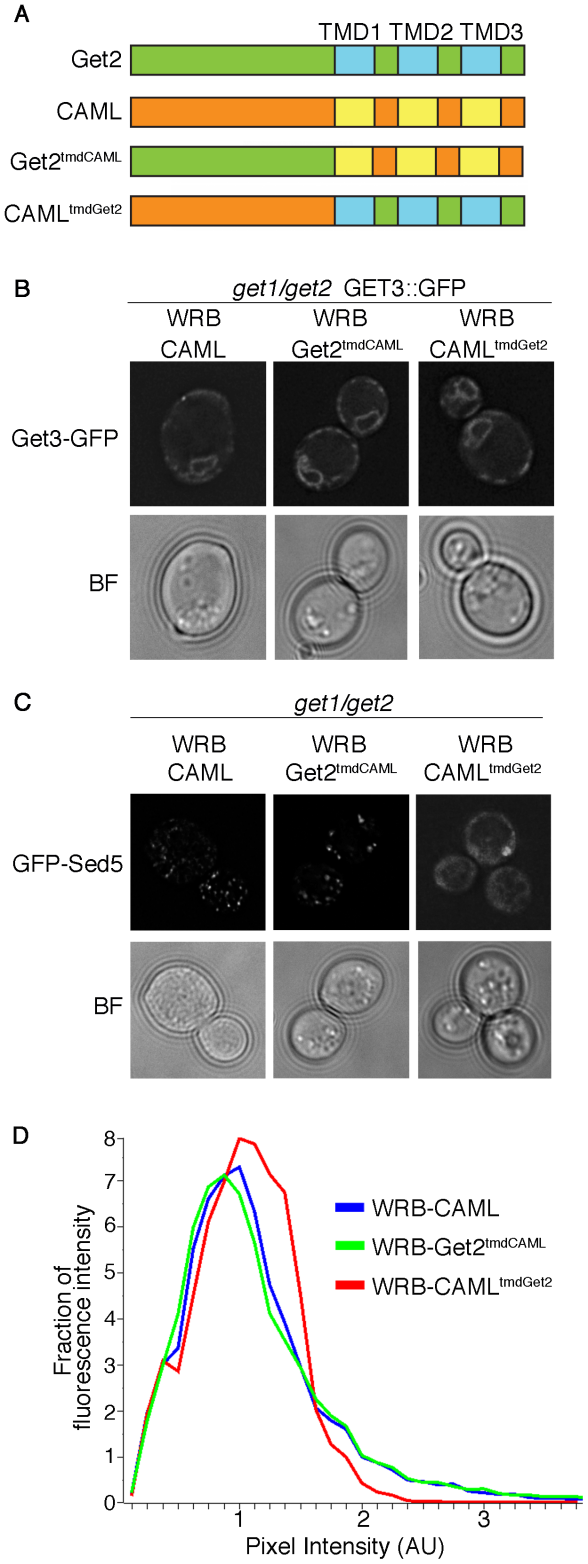
The transmembrane domains of CAML are essential for a functional WRB/CAML receptor complex. (A) Schematic representation of CAML-Get2 chimeras. Position of transmembrane domains (TMD) are indicated. (B) *get1/get2* yeast cells carrying a genomically GFP-tagged version of Get3 were transformed with a plasmid containing the coding sequence of WRB in combination with CAML or CAML-Get2 chimeras. Subcellular Get3-GFP localization was analyzed by fluorescence microscopy. (C) *get1/get2* yeast cells were transformed with a plasmid containing the coding sequence of GFP-tagged Sed5 and Get1/Get2 or WRB in combination with CAML or CAML-Get2 chimeras. Subcellular GFP-Sed5 localization was analyzed by fluorescence microscopy. (D) Images taken in (C) were quantified to determine the distribution of fluorescence across bins of different pixel intensity for each strain. A minimum of 42 cells was analyzed per strain.

We have demonstrated that CAML is much more efficient than Get2 in creating a functional receptor together with WRB. A similar topology was suggested for Get2 and CAML. Both proteins are predicted to have a cytosolically exposed N-terminal domain and three TMDs at the C-terminus [6], [11] (Figure 3A). In order to investigate the differences between Get2 and CAML, we generated Get2-CAML chimeric proteins in which the TMDs were swapped (Figure 3A). The resulting Get2^tmdCAML^ and CAML^tmdGet2^ were cotransformed with a plasmid encoding WRB into *get1/get2* cells, where they expressed robustly. Interestingly, WRB had a stabilizing effect on Get2^tmdCAML^ (as previously observed for CAML; Figure S1C) but not on Get2 or CAML^tmdGet2^, which expressed equally well in the absence of WRB (Figure S4). We co-expressed a GFP-tagged version of Get3 and localization of Get3-GFP was analyzed by fluorescence microscopy. In both cases, Get3 was efficiently recruited to the ER membrane (Figure 3B) suggesting that both chimeras can contribute to providing Get3-binding sites at the ER when coexpressed with WRB.

To analyze the ability of the chimeras to mediate TA protein insertion, we transformed them into *get1/get2* cells together with WRB and a GFP-tagged version of Sed5. Interestingly, only Get2^tmdCAML^ allowed proper targeting of GFP-Sed5, whereas in presence of CAML^tmdGet2^, Sed5 remained predominantly cytosolic or in punctate structures characteristic of the deposition sites for aggregated proteins (Figure 3C). These data were confirmed by pixel fluorescence intensity measurement (Figure 3D): The complementation by WRB-CAML and WRB-Get2^tmdCAML^ is reflected in a shift of the peak to the left (reduction in the number of pixels of moderate intensity found in the cytosolic background) and the presence of a shoulder representing bright punctate fluorescence in Golgi structures. Furthermore, in combination with WRB, the Get2^tmdCAML^ chimera actively supported the ER membrane insertion of Sbh2 (Figure S3). Cells expressing WRB in combination with Get2^tmdCAML^, but not with CAML^tmdGet2^, could also grow in presence of several agents causing oxidative or protein folding stress (Figure S5). In fact, the Get2^tmdCAML^ chimera was functionally indistinguishable from CAML when expressed in combination with WRB (Figure 3D, S5). However, there is no evidence for any function of the inverse chimera CAML^tmdGet2^ in conjunction with WRB – it did not support targeting of either TA protein (Figure 3, S3) and failed to rescue growth under any of the stress conditions (Figure S5). This demonstrates that a matching set of TMDs is required for forming a functional GET receptor but leaves open whether this region is required to correctly position the cytosolic domains of WRB and CAML or whether it plays an active role during TA targeting.

To investigate this further, we performed a split-ubiquitin based yeast two-hybrid assay and tested whether WRB can create a complex with Get2 or with Get2-CAML chimeric proteins. WRB bait construct was transformed into NMY51 yeast cells together with the respective prey constructs and protein expression was monitored by immunoblot analysis (Figure S6A). Cell growth on selective plates was observed only when WRB was expressed with CAML or Get2^tmdCAML^ demonstrating that WRB and CAML form a functional TRC40 receptor complex via the interaction of their transmembrane segments as previously shown [10]. This analysis provides no evidence for complex formation between WRB and Get2 or CAML^tmdGet2^– in fact, Get2 and CAML^tmdGet2^are not affected by coexpression of WRB (Figure S4B). Since lower-affinity interactions may escape detection by this assay, we cannot exclude that a WRB/Get2 complex may form transiently.

We conclude that the combined cytosolic portions of Get1/Get2, WRB/CAML or WRB/Get2 can recruit Get3 to the ER membrane – independently of a stable complex being formed between the membrane proteins. Coexpression of WRB and Get2 may simply increase the number of Get3 docking sites on the ER membrane above a threshold required for the localization to be visible by microscopy. However, a matching heteromultimeric transmembrane assembly comprising either the transmembrane domains of Get1/Get2 or WRB/CAML is clearly required to form a fully functional receptor with high TA protein targeting activity. The results presented in this study suggest that targeting Get3 to the ER membrane is not sufficient to ensure TA protein insertion. A matching TMD assembly of the GET receptor may either be required to coordinate the interactions between Get3 and the cytosolic receptor domains by properly orienting them or may indeed be actively involved in the membrane insertion process.

We showed that CAML is not a functional Get2 equivalent because it couldn’t form a functional receptor with Get1. However, together with the Get1 orthologue WRB, CAML takes on a role analogous to Get2 in the context of the mammalian ER membrane. This results in the formation of a protein complex that can fully replace the yeast receptor (Table 1).

**Table 1:** Summary of the results obtained in this study.

Transformation	Interaction	Growth rescue	Get3 membrane	TA protein
in NMY51 or *get1/get2* cells	assayed by splUb2H		targeting	insertion
Mock	No	no	no	no
Get1/Get2	n.a.	yes	yes	yes
WRB	–	no	no	no
CAML	–	no	no	no
WRB/CAML	Yes	yes	yes	yes
WRB/Get2	No	yes	yes	partial
CAML/Get1	n.a.	no^1^	no	no
WRB/CAML^tmdGet2^	No	no	yes	no
WRB/Get2^tmdCAML^	Yes	yes	yes	yes

^1^ growth rescue at 39°C.

‘n.a.’ = not assayed’.

The mechanism by which a TA protein is finally integrated into the ER membrane is still elusive. Different hypotheses have been proposed [23]: the insertion may occur spontaneously once the TA protein is in close proximity of the membrane or the receptor complex may assist the integration. Structural analysis of the cytosolic domains of Get1 and Get2 has provided mechanistic evidence for an active role in TA protein release [7], [8]. However, how the transmembrane region of the receptor may interact with the TA protein substrate remains unclear. Our results demonstrate an important role of a matching set of TMDs contributed by either Get1/Get2 or WRB/CAML. This region is clearly required to ensure stable complex formation but may also functionally contribute by orienting the cytosolic domains or by interacting with the TA substrate during the membrane integration process.

To complement our *in vivo* characterization of WRB and CAML, we aimed to quantitatively compare the binding parameters of TRC40 to the cytosolic domains of WRB and CAML in isolation by surface plasmon resonance (SPR). Furthermore, we used reflectometric interference spectroscopy (RIfS) to thermodynamically and kinetically analyze the binding of TRC40 to its membrane receptors in the context of a native mammalian microsomal membrane. RlfS can be used to monitor the interaction of proteins with lipid membranes via changes in optical thickness [24]. For the RIfS measurement, we spread canine rough microsomes together with unilamellar 1-palmitoyl-2-oleoyl-*sn*-glycero-3-phosphocholine (POPC) liposomes on a silicon-SiO_2_ chip to form a planar and defect-free lipid bilayer serving as a mimic of the ER membrane. The success of the preparation was controlled by fluorescence microscopy using Texas Red 1,2-dihexyldecanoyl-sn-glycero-3-phosphoethanolamine (DHPE; 1 mol% in POPC vesicles) as a membrane label. The SiO_2_ serves as the transducer film to create interference fringes that permit detection of changes in optical thickness due to protein adsorption. Increasing concentrations of wild type TRC40 either unloaded or in complex with the substrate TA protein RAMP4 [25] were passed over the surface (Figure S7A and B) and monitored by an optical readout system comprising a fiber to illuminate the sample with white light, and a bundle of light fibers for spectral readout. The adsorption isotherm was fitted with a Langmuir equation providing an affinity constant (K_D_) of 251 nM for TRC40 and 262 nM for the TRC40/RAMP4 complex, respectively (Table 2). Binding of TRC40 and TRC40/RAMP4 complex to the microsomal membrane resulted in an increase in optical thickness of the membrane, whereas we could not record any change in optical thickness of a biosensor coated with pure lipids (POPC) (Figure S7C) in agreement with the necessity of a receptor protein complex for TRC40 binding to the membrane.

**Table 2:** Reflectometric Interference Spectroscopy (RIfS) and Surface plasmon resonance (SPR) measurements.

Method	Ligand	Analyte	K_D_	± St. Err.
**RIfS**	Rough Microsomes	TRC40	251 nM	65 nM
		TRC40-R4	262 nM	137 nM
**SPR**	WRBcc	TRC40	392 nM	30 nM
		TRC40-R4	402 nM	37 nM
	CAMLcyt	TRC40	85 nM	29 nM
		TRC40-R4	34 nM	6 nM

Affinity constants of TRC40 and TRC40/RAMP4 complex for rough microsomes, WRBcc and CAMLcyt. Values represent the average calculated from three independent measurements.

To obtain the binding affinity constants of TRC40 for each cytosolic component of the receptor complex, the coiled coil domain of WRB (WRBcc) [9] and the cytosolic domain of CAML (CAMLcyt) were immobilized on a SPR sensor chip, and association and dissociation of TRC40 were followed over time. The results summarized in Table 2 (and binding isotherms from representative experiments shown in Figure S8) indicate a higher affinity of TRC40 for CAML than for WRB, either unloaded or in complex with RAMP4. It is therefore conceivable that CAML represents the first docking site for TRC40 at the ER membrane prior to an interaction of TRC40 with WRB. Similarly, it has been proposed that yeast Get2 may act as a tethering protein for Get3 at the ER membrane before its stable association with Get1 in a next step of the targeting process [7].

SPR provides affinity constants for single protein-protein interaction, whereas RIfS allowed us to measure the affinity of TRC40 for the full receptor complex in its physiological membrane environment. As the values obtained from the two systems are in a similar range, we conclude that, as already shown for yeast Get1 and Get2 reconstituted in proteoliposomes [8], receptors formed by WRB and CAML can satisfactorily explain recruitment of TRC40 to the ER membrane. Taken together with the functional complementation of a *get1/get2* mutant by WRB/CAML in yeast, this result suggests that the mammalian receptor does not contain additional essential subunits for its basic function.

CAML has been shown to affect highly specialized biological processes in terminally differentiated cells such as immune cells or inner hair cells [11], [16]. In analogy to the pleiotropic phenotypes of yeast cells lacking the GET system, it is difficult to decide whether all CAML-related observations will eventually be explained by effects on TA protein targeting or whether the protein fulfills several independent functions. A recent report links mammalian TA protein targeting to calcium signaling via Ca^2+^-calmodulin [26]. With its clear links to calcium signaling, further dissection of CAML function may provide an avenue to better understand the physiological regulation of TA protein biogenesis by calcium. Functional complementation in yeast is a classical approach to demonstrating functional equivalence of proteins and enables detailed structure-function studies. Specifically, *get1/get2* deletion in yeast can now be used to dissect the functionally important domains for the WRB/CAML interaction, for Get3 binding and for TA protein insertion in the absence of endogenous WRB or CAML.

## Materials and Methods

### Yeast Strains and Growth Conditions

*S. cerevisiae* strain NMY51 (*MATa his3delta200 trp1–901, leu2–3,112 ade2, LYS2::(lexAop)_4_-HIS3 ura3::(lexAop)_8_-lacZ (lexAop)_8_-ADE2 GAL4*) for split ubiquitin yeast two hybrid was obtained from Dualsystem Biotech. *get1/get2*, *get1* GET3::GFP, *get2* GET3::GFP and *get1/get2 GET3*::GFP strains were previously described [3].

Cells were grown in Hartwell’s Complete (HC) medium. All experiments were performed at mid-log phase.

### Plasmids

Constructs used in this study were obtained by standard cloning procedures [27] and verified by sequencing. p416MET25-Get1–8PC10his and BFGIII-Get2 for yeast expression of Get1 and Get2 respectively were described in [3]. pQE80-MBP-TRC40wt and pT5L/T7-MBP-TRC40wt/HZZ-R4op for bacterial expression of MBP-TRC40 and MBP-TRC40/HZZ-RAMP4op protein complex were described in [25]. pQE80-MBP-WRBcc for bacterial expression of the coiled-coil domain of WRB was described in [9].

For p416MET25-WRB, used for yeast expression of WRB, the coding sequence of WRB was amplified from pCMW-Sport6-WRB (IMAGE consortium, Berlin) using the primers TATCTA AGATCTGCCACCATGAGCTCAGCCGCG and TAGATAGAATTCTCAGCTGAACGGATGAAG containing BglII and EcoRI restriction sites respectively. The fragment was then cloned into p416MET25 backbone [28].

For p416MET25-WRB-Cub-LexA, used for expression of WRB in NMY51 yeast strain and yeast two hybrid experiments, the coding sequence of WRB was amplified from pCMW-Sport6-WRB using the primers GCTCTAGAGACCATGAGCTCAGCCGCG and ATAAGTGGCGGAGGCGGCCAAGCTGAACGGATGAAGCAC containing XbaI and SfiI restriction sites respectively. The fragment was cloned into pDHB1 vector (Dualsystem Biotech). A XbaI/EcoRI fragment originated from pDHB1-WRB was then subcloned into the p416MET25 backbone.

For pPR3-CAML, used for expression of CAML in NMY51 yeast strain and yeast two hybrid experiments, the coding sequence of CAML was amplified from pCMV-Sport6-CAML (IMAGE consortium, Berlin) using the primers TATATTGGCCATTACGGCCATGGAGTCGATGGCCGTC and TTAATAGGCCGAGGCGGCCTCATGGTACTTCAGAGCC both containing SfiI restriction sites. The fragment was cloned into the pPR3-N backbone (Dualsystem Biotech).

For p415MET25-CAML, used for yeast expression of CAML, a SpeI/EcoRI fragment from pPR3-CAML was subcloned into the p415MET25 backbone [28] This construct carries a HA epitope from the pPR3 backbone.

For pGEX-CAMLcyt, used for bacterial expression of the cytosolic domain of CAML, the coding sequence of CAML cytosolic domain was amplified from pCMV-Sport6-CAML using the primers ATACTAGAATTCATGGAGCCGGTGCCT and TACATACTCGAGTCATCGAAAG containing EcoRI and XhoI restriction sites respectively. The fragment was cloned into the pGEX-5X-1 backbone (Amersham).

For p415MET25-CAMLtmdGet2, used for yeast expression of CAML^tmdGet2^, the coding sequence of CAML cytosolic domain was amplified from pPR3-CAML using the primers CCATACTCTAGAACTAGTATGCAGATTTTC and TAGTATCTGCAGCCAATCGAAATATTCGAAAAGAGTC containing XbaI and PstI restriction sites respectively. The fragment was cloned into p415MET25-Get2–4PC [3]. This construct carries a HA epitope from the pPR3 backbone.

For p415MET25-Get2tmdCAML, used for yeast expression of Get2^tmdCAML^, the coding sequence of Get2 cytosolic domain was amplified from p415MET25-Get2–4PC using the primers TATCTAGAATGTACCCATACGATGTTCCAGATTACGCTATGTCTGAATTAACAGAGGCGG and TAGTATCCGCGGGTCTGTTCAGCAAGTAATCAATG containing XbaI and SacII restriction sites respectively. The fragment was cloned into p415MET25-CAML. A HA epitope was introduced in the forward primer.

For pPR3-Get2, used for expression of Get2 in NMY51 yeast strain and yeast two hybrid experiments, a BamHI/EcoRI fragment from p415MET25-Get2–4PC was subcloned into the pPR3-N backbone.

For pPR3-Get2tmdCAML, used for expression of Get2^tmdCAML^ in NMY51 yeast strain and yeast two hybrid experiments, a BamHI/EcoRI fragment from p415MET25-Get2tmdCAML was subcloned into the pPR3-N backbone.

For pPR3-CAMLtmdGet2, used for expression of CAML^tmdGet2^ in NMY51 yeast strain and yeast two hybrid experiments, a BamHI/EcoRI fragment from p415MET25-CAMLtmdGet2 was subcloned into the pPR3-N backbone.

pRS413-GFP-Sed5, for yeast expression of GFP-Sed5 was described in [17].

For p413-CYC1-cherry-Sbh2, used for yeast expression of cherry-Sbh2, a SpeI/ClaI fragment from p415-cherry-Sbh2 [3] was subcloned into the p413-CYC1 backbone [29].

### Yeast Two-hybrid Analysis

To verify the interaction between WRB and CAML, the split ubiquitin yeast two hybrid method was used [30].

The WRB bait construct p416-Cub-WRB-LexA was transformed into NMY51 *S. cerevisiae* cells with the empty prey vector pPR3-N, or constructs for expression of N-ubiquitin-tagged versions of CAML, Get2 and CAML-Get2 chimeric proteins. Co-transformants were selected on HC-ura-trp plates. Colonies were inoculated over night in liquid HC-ura-trp medium, diluted to an OD600 of 0.2 and 1∶5 serial dilutions spotted on HC-ura-trp or HC-ura-trp-his plates.

### Fluorescence Microscopy

Images of live cells were acquired at room temperature on a Delta Vision RT (Applied Precision) microscope using a 100×/0.35–1.5 Uplan Apo objective and specific band pass filter sets for GFP. The images were collected using a Coolsnap HQ (Photometrics) camera. Image processing was performed using ImageJ (http://rsbweb.nih.gov/ij/). Pixel fluorescence intensity of at least 20 fields per sample (see cell number in Figure Legend) was quantified as described in [17], [21] using Knime software (www.knime.org/knime).

### Western Blotting

1 ml of mid-log phase yeast culture was pelleted by low speed centrifugation and resuspended in 0.1 M NaOH. Cells were recovered by centrifugation and dissolved in 1X SDS loading buffer. 10 µl of lysate per lane were separated by SDS-PAGE and analyzed by immunoblot using mouse monoclonal against HA epitope (Sigma) or Pgk1 (Molecular Probes), or rabbit polyclonal antibodies against LexA (Millipore), PDI-1 [17], WRB and CAML [10]. For densitometric analysis, ImageJ software was used.

### Protein Expression and Purification

MBP-TRC40 and MBP-WRBcc expression was induced in BL21AI *E. coli* strain with 1 mM IPTG for 2 hours at 30°C. Cells were then harvested and resuspended in ice-cold LS buffer (50 mM HEPES, 150 mM KOAc, 10 mM MgOAc_2_, 10% glycerol, 1 mM PMSF, pH 7.0) containing 10 µg/ml DNase I. After lysis with Avestin Emulsiflex-C5 aggregates were removed by centrifugation at 100,000 *g*, 30 minutes, 4°C. The supernatant was loaded onto an amylose resin (NEB) column. The resin was washed with 10 volumes of LS buffer containing 5 mM ATP, 10 volumes of HS buffer (50 mM HEPES, 500 mM KOAc, 10 mM MgOAc_2_, 10% glycerol, 1 mM PMSF, pH 7.0) and 10 volumes of LS buffer. Proteins were eluted with LS buffer containing 20 mM maltose [9].

To remove the N-terminal tag from MBP-WRBcc, the protein was incubated with histidine tagged TEV protease in a 1∶30 w/w ratio for 2 hours at 4°C. TEV protease was removed by incubation with Ni-NTA resin (Qiagen).

For purification of the MBP-TRC40/HZZ-RAMP4op complex, MBP-TRC40 was first induced with 0.05 mM IPTG for 1 hour, then HZZ-RAMP4op was induced with 0.5% arabinose for 4 hours. Cells were then harvested and resuspended in ice-cold HS buffer (50 mM HEPES, 500 mM NaCl, 10 mM MgCl_2_, 10% glycerol, 1 mM PMSF, pH 7.0) containing 40 mM imidazole and 10 µg/ml DNase I, lysed using Avestin Emulsiflex-C5 and aggregates pelleted by centrifugation for 30 minutes at 100,000 *g*. The supernatant was loaded onto a Ni-NTA resin (Qiagen), washed with HS buffer containing 40 mM imidazole and proteins were eluted with HS buffer containing 500 mM imidazole. Eluted samples were loaded onto an amylose resin column (NEB) and the column washed with HS buffer, LSATP buffer (50 mM HEPES, 150 mM NaCl, 10 mM MgCl_2_, 10 mM ATP, 10% glycerol, 1 mM PMSF, pH 7.0) and HS buffer containing 10 mM arginine and 1 mM DTT. Proteins were eluted with HS buffer containing 20 mM maltose [25].

The GST-tagged cytosolic domain of CAML was expressed in BL21-AI *E. coli* strain by induction with 0.5 mM IPTG at 30°C for 3 hours. Cells were harvested and resuspended in ice cold PBS containing 1 mM PMSF and 10 µg/ml DNase I. After lysis with Avestin Emulsiflex-C5, aggregates were removed by centrifugation at 100,000 x g for 30 minutes at 4°C. Bacterial lysate was loaded onto a Protino Glutathione agarose 4B (Machinery-Nagel) column. The resin was washed with 10 volumes PBS. Proteins were eluted with 10 volumes of GST elution buffer (50 mM Tris base, 10 mM glutathione, 1 mM PMSF, pH 8.0).

### Surface Plasmon Resonance (SPR)

SPR measurements were performed using a Reichert SPR Biosensor SR7000DC with a SPR sensorchip HC1000m (XanTec bioanalytics), in PBS, at 15°C, at a flow rate of 40 µl/min. WRBcc or CAMLcyt were immobilized on the left (sample) channel of the chip at a concentration of 600 nM with a flow rate of 30 µl/min to response unit (RU) levels of 5000–6000 RU or 20000–25000 RU respectively. The right channel served as a reference. Increasing concentrations of analyte (TRC40wt or TRC40wt/RAMP4) in PBS were injected for 270 s to both channels, and dissociation was followed for 12 min. The difference in response between sample and reference channels was recorded. Affinity and kinetic data analysis was performed using Scrubber 2.0 (BioLogic Software). The response was referenced to blank (buffer) injections and normalized using the molecular weight of the ligand (in kDa).

### Reflectometric Interference Spectroscopy (RIfS)

A silicon chip with a 5micron thick layer of SiO_2_ was used as RIfS transducer chip. The silicon chip was cleaned prior to measurement with ammoniac piranha solution (NH_3_/H_2_O_2_/H_2_O, 1/1/5) at 70°C for 15 min and activated via O_2_ plasma for 1 min. After 10 minutes, a microsome-liposome mixture was added to the RIfS system (NanoCalc-2000-UV/Vis/NIR spectrometer, Ocean Optics, Ostfildern, Germany) and membrane formation was monitored leading to a change in optical thickness of about 6–8 nm. Excess vesicle solution was washed out of the system with buffer and once the membrane signal stabilized, the measurement of adsorption isotherms was commenced. To determine the affinity of the protein to its membrane receptor, the protein concentration, was increased stepwise (40 nM to 1 µM) and the system was left to equilibrate after each protein addition. By plotting the signal increase against the concentration, we could determine a dissociation constant K_D_ for the protein-receptor complex by applying a Langmuir fit. At the end of each measurement, the whole system was washed with buffer for at least 30 min.

### Preparation of Reconstituted Cellular Membranes for RIfS Measurements

Lipid films of pure 1-palmitoyl–2-oleoyl-*sn*-glycero-3-phosphocholine (POPC; purchased from Avanti Polar Lipids, Inc., Alabaster, Alabama, USA) were prepared in a test-tube by drying a chloroform solution of the lipid (0.25 mg of lipid per film). The reconstituted cellular membranes were prepared as previously described [31]. A lipid film was left to swell in buffer solution (50 mM HEPES, 100 mM KAc, 1 mM MgAc, pH 7.0) in a waterbath at 50°C for 15 min. Microsomes were added to the POPC vesicle in a weight ratio of 3/2 yielding a concentration of 1 mg/ml. The microsome-liposome suspension was extruded 40 times through a porous polycarbonate membrane (pore diam.: 400 nm) at room temperature.

## Supporting Information

Figure S1 **WRB and CAML interact in yeast cells and stabilize each other.** (A) Yeast cells (NMY51 strain) were transformed with p416-Cub-WRB-LexA in combination with pPR3 (mock) or pPR3-CAML for split-ubiquitin yeast two hybrid analysis. Serial dilutions were spotted on HC-ura-trp or HC-ura-trp-his. (B) *get1/get2* yeast cells were transformed with WRB alone or in combination with Get2 or CAML. Protein lysates were separated by SDS-PAGE and analyzed by immunoblot for Pdi1 as a loading control and WRB. The graph shows the relative expression of WRB. Error bars indicate standard error calculated from four independent experiments. ***: p<0.0001; *: p<0.05. (C) *get1/get2* yeast cells were transformed with CAML alone or in combination with Get1 or WRB. Protein lysates were separated by SDS-PAGE and analyzed by immunoblot for Pdi1 and CAML. The graph shows the relative expression of CAML. Error bars indicate standard error calculated from four independent experiments. **: p<0.001; *: p<0.05; *ns*: not significant.
(TIF)

Figure S2 **Get3-GFP localization in** ***get1*** **and** ***get2*** **yeast cells.** (A) *get1* yeast cells carrying a genomically GFP-tagged version of Get3 were transformed with an empty vector or vectors containing the coding sequence of Get1 or WRB. Get3-GFP localization was recorded by fluorescence microscopy. (B) *get2* yeast cells carrying a genomically GFP-tagged version of Get3 were transformed with an empty vector or vectors containing the coding sequence of Get2 or CAML. Get3-GFP localization was recorded by fluorescence microscopy. Note that the partner subunit (Get2 in Figure S2A and Get1 in Figure S2B) is present at endogenous levels in Figure S2 in the single deletion strains, whereas it was expressed from the same plasmid and promoter as the tested construct in the double deletion strain shown in Figure 2A. This may explain a higher cytosolic pool of Get3-GFP, in addition to a clearly visible ER-localized pool, in Figure S2.
(TIF)

Figure S3 **In combination with CAML or Get2^tmdCAML^, WRB rescues ER membrane insertion of Sbh2.** *get1/get2* yeast cells were transformed with a plasmid containing the coding sequence of cherry-tagged Sbh2 and combinations of constructs encoding WRB, CAML, Get1, Get2 and Get2-CAML chimeras. Subcellular cherry-Sbh2 localization was analyzed by fluorescence microscopy.
(TIF)

Figure S4 **Effect of WRB expression on CAML, Get2 and CAML-Get2 chimeric proteins.** (A) *get1/get2* cells were transformed with vectors containing the coding sequence of CAML, Get2 or CAML-Get2 chimeric proteins either alone or in combination with a WRB encoding construct. Proteins were detected by immunoblot analysis using an antibody against the HA epitope. Pgk1 was analyzed as a loading control. (B) Expression of CAML, Get2 and CAML-Get2 chimeric proteins was normalized to the loading control and relative quantification compared to cells not transformed with WRB is shown in the graph. Data were calculated from four independent experiments. Error bars indicate standard error. *: p<0.0001; *ns*: not significant.
(TIF)

Figure S5 **The transmembrane domains of CAML are required to rescue the growth phenotypes of** ***get1/get2*** **yeast cells.** *get1/get2* yeast cells were transformed with Get1 and Get2 encoding constructs or WRB in combination with CAML or CAML-Get2 chimeric constructs and serial dilutions spotted on different conditions: HC plates incubated at 30°C (control), 37°C+CuSO_4_, 39°C, H_2_O_2_, hydroxyurea, tunicamycin, hygromycin.
(TIF)

Figure S6 **WRB and CAML interact in yeast cells via their transmembrane segments.** (A) Yeast cells (NMY51 strain) were transformed with p416-Cub-WRB-LexA in combination with pPR3 (mock), pPR3-CAML, pPR3-CAMLtmdGet2, pPR3-Get2 or pRP3-Get2tmdCAML for split-ubiquitin yeast two-hybrid analysis. Serial dilutions were spotted on HC-ura-trp or HC-ura-trp-his. (B) Protein lysates from NMY51 yeast cells used in split-ubiquitin yeast two-hybrid were separated by SDS-PAGE and analyzed by immunoblot with anti-LexA and anti-HA antibodies.
(TIF)

Figure S7 **Analysis of TRC40 binding to its membrane receptor complex by Reflectometric Interference Spectroscopy (RIfS).** (A) Schematic representation of a RIfS measurement. A mixture of canine pancreatic rough microsomes and POPC liposomes is spread over a silicon-SiO_2_ chip. Analytes (TRC40 or TRC40-R4) are then injected. Increase in optical thickness (OT) is followed over time. (B) Representative RIfS measurement. Arrows indicate injection of increasing concentration of TRC40 (from 40 nm to 1 µM) and a final buffer washing step. The curve shows variations in OT over time. (C) Final increase in OT at the end of RIfS measurements for experiment performed in presence of microsomes (RM) or pure lipid layers (POPC).
(TIF)

Figure S8 **Kinetic characterization of WRBcc and CAMLcyt binding to TRC40.** Binding isotherms for TRC40 binding to WRBcc or CAMLcyt calculated by Surface Plasmon Resonance.
(TIF)
