# Supplementary figures and images for: WRB and CAML Are Necessary and Sufficient to Mediate Tail-Anchored Protein Targeting to the ER Membrane
Source: PLoS One. 2014 Jan 2;9(1):e85033. doi: 10.1371/journal.pone.0085033 (PMC3879356; doi:10.1371/journal.pone.0085033)

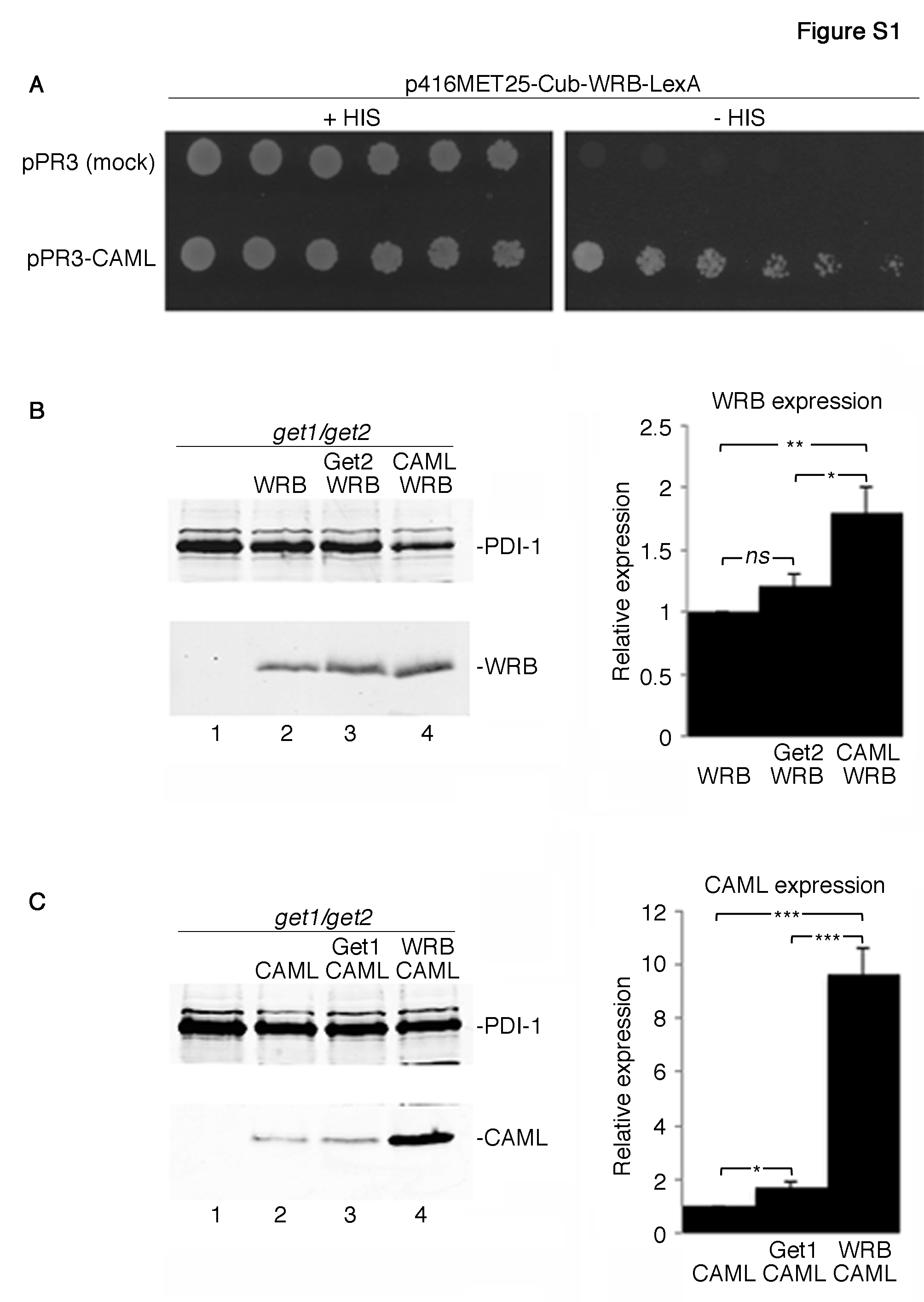

Supplement: Figure S1 — WRB and CAML interact in yeast cells and stabilize each other. (A) Yeast cells (NMY51 strain) were transformed with p416-Cub-WRB-LexA in combination with pPR3 (mock) or pPR3-CAML for split-ubiquitin yeast two hybrid analysis. Serial dilutions were spotted on HC-ura-trp or HC-ura-trp-his. (B) get1/get2 yeast cells were transformed with WRB alone or in combination with Get2 or CAML. Protein lysates were separated by SDS-PAGE and analyzed by immunoblot for Pdi1 as a loading control and WRB. The graph shows the relative expression of WRB. Error bars indicate standard error calculated from four independent experiments. ***: p<0.0001; *: p<0.05. (C) get1/get2 yeast cells were transformed with CAML alone or in combination with Get1 or WRB. Protein lysates were separated by SDS-PAGE and analyzed by immunoblot for Pdi1 and CAML. The graph shows the relative expression of CAML. Error bars indicate standard error calculated from four independent experiments. **: p<0.001; *: p<0.05; ns: not significant. (TIF) [file pone.0085033.s001.tif]

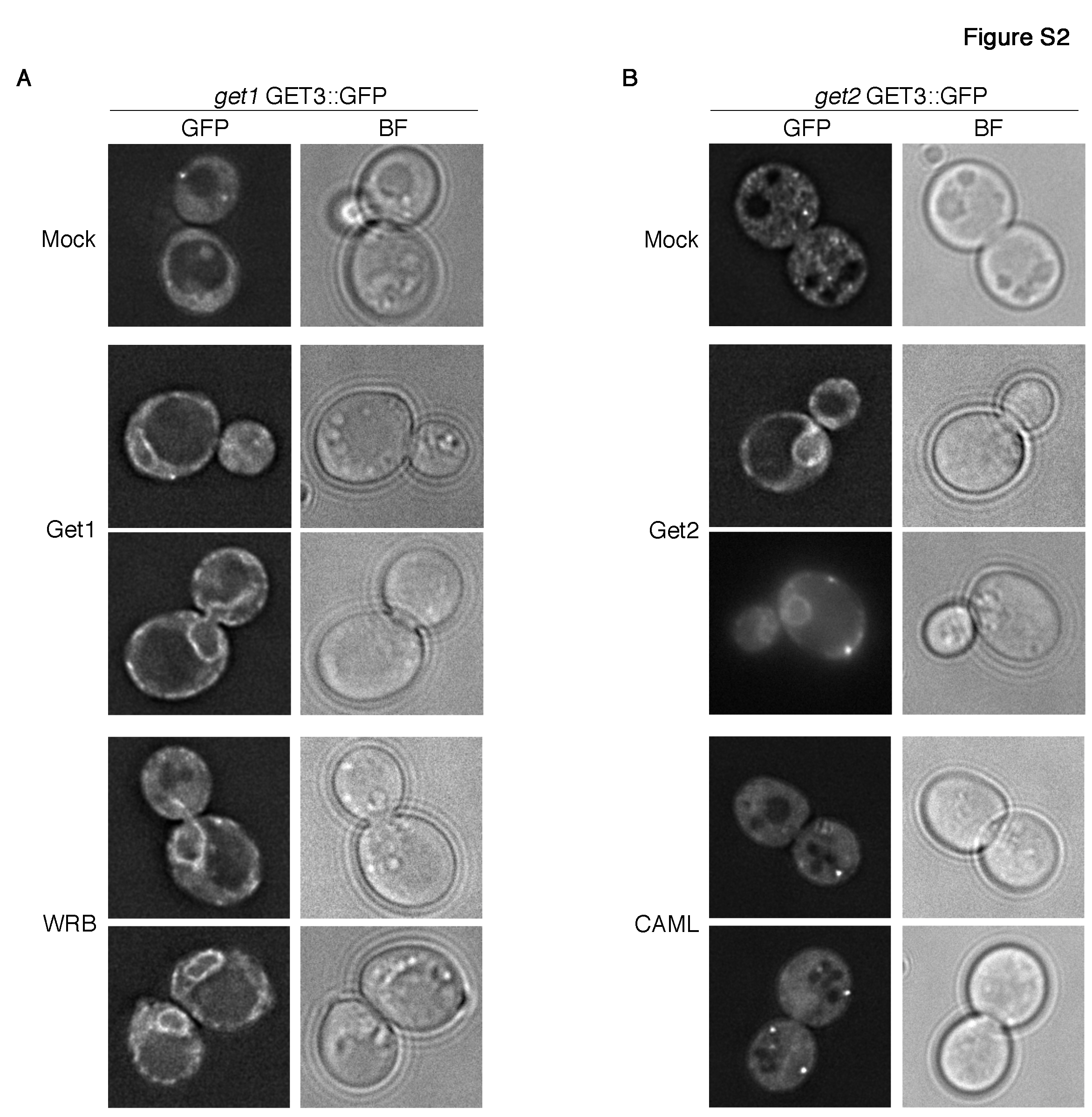

Supplement: Figure S2 — Get3-GFP localization in get1 and get2 yeast cells. (A) get1 yeast cells carrying a genomically GFP-tagged version of Get3 were transformed with an empty vector or vectors containing the coding sequence of Get1 or WRB. Get3-GFP localization was recorded by fluorescence microscopy. (B) get2 yeast cells carrying a genomically GFP-tagged version of Get3 were transformed with an empty vector or vectors containing the coding sequence of Get2 or CAML. Get3-GFP localization was recorded by fluorescence microscopy. Note that the partner subunit (Get2 in Figure S2A and Get1 in Figure S2B) is present at endogenous levels in Figure S2 in the single deletion strains, whereas it was expressed from the same plasmid and promoter as the tested construct in the double deletion strain shown in Figure 2A. This may explain a higher cytosolic pool of Get3-GFP, in addition to a clearly visible ER-localized pool, in Figure S2. (TIF) [file pone.0085033.s002.tif]

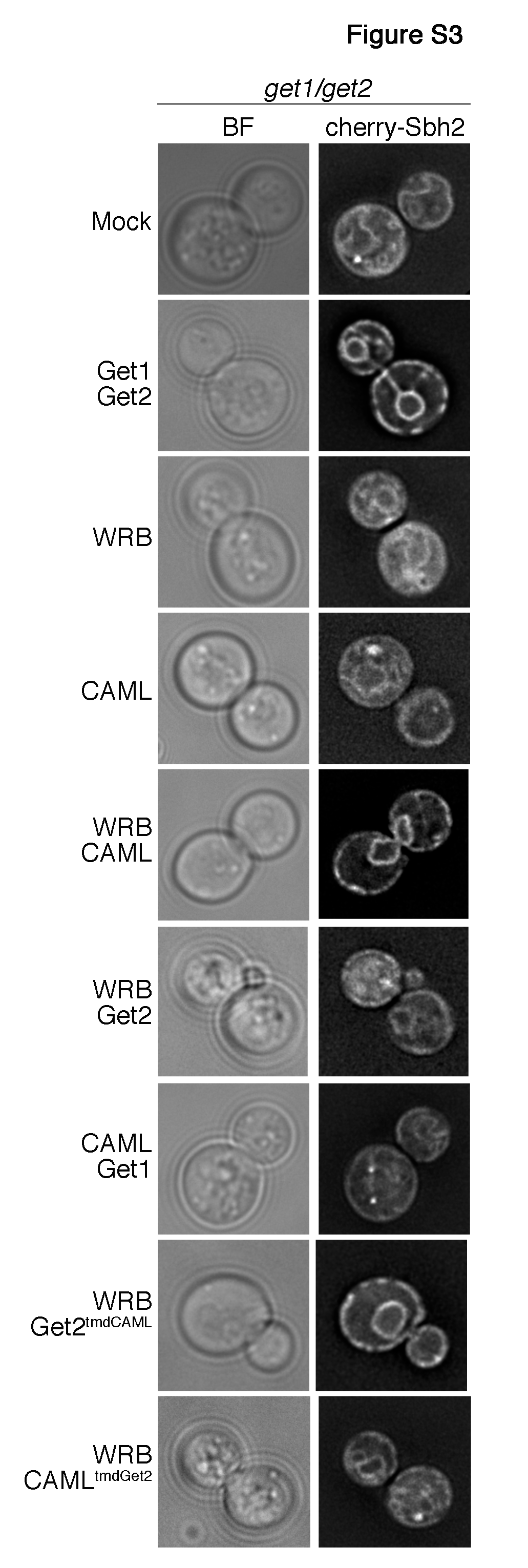

Supplement: Figure S3 — In combination with CAML or Get2tmdCAML, WRB rescues ER membrane insertion of Sbh2. get1/get2 yeast cells were transformed with a plasmid containing the coding sequence of cherry-tagged Sbh2 and combinations of constructs encoding WRB, CAML, Get1, Get2 and Get2-CAML chimeras. Subcellular cherry-Sbh2 localization was analyzed by fluorescence microscopy. (TIF) [file pone.0085033.s003.tif]

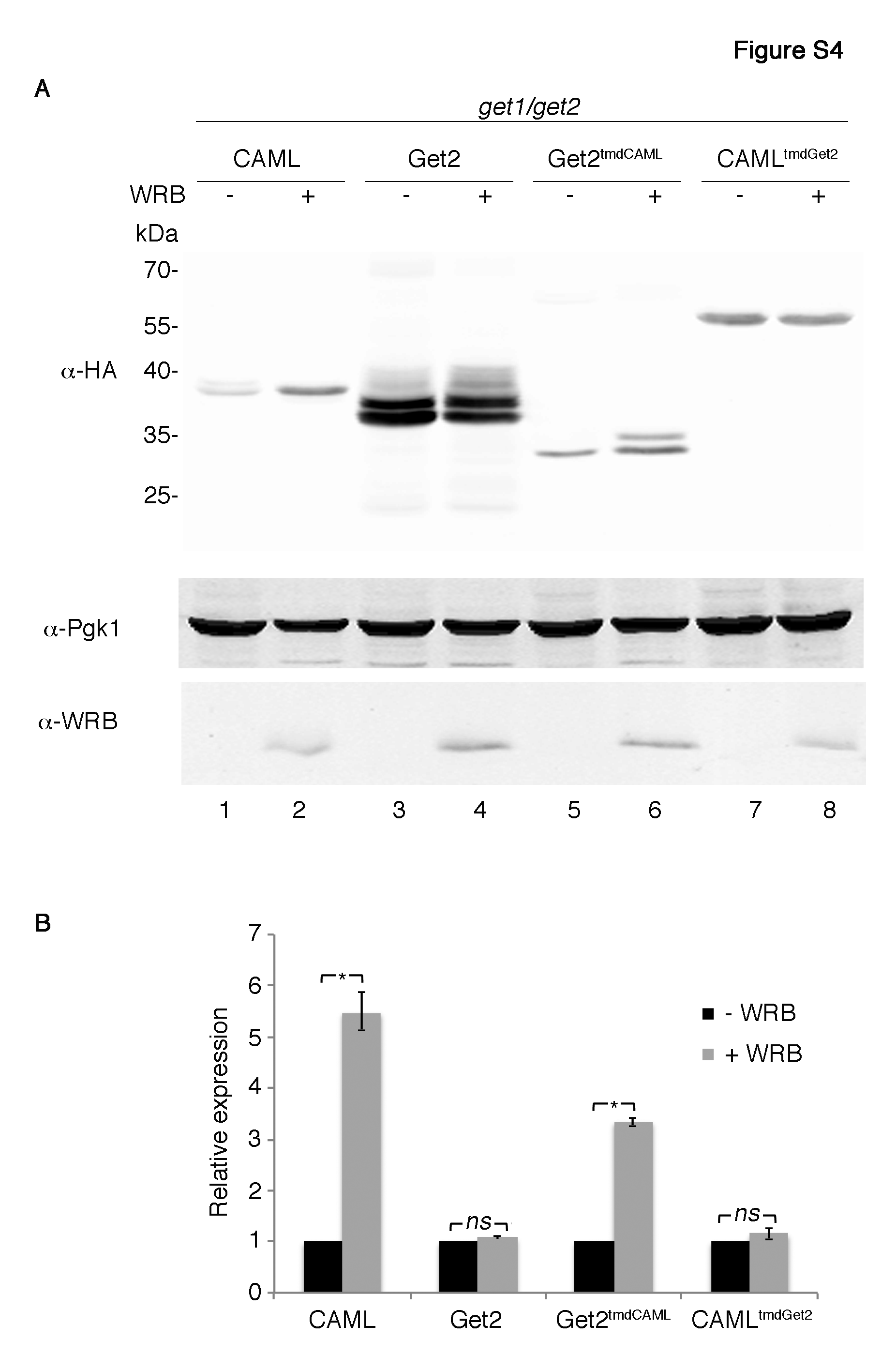

Supplement: Figure S4 — Effect of WRB expression on CAML, Get2 and CAML-Get2 chimeric proteins. (A) get1/get2 cells were transformed with vectors containing the coding sequence of CAML, Get2 or CAML-Get2 chimeric proteins either alone or in combination with a WRB encoding construct. Proteins were detected by immunoblot analysis using an antibody against the HA epitope. Pgk1 was analyzed as a loading control. (B) Expression of CAML, Get2 and CAML-Get2 chimeric proteins was normalized to the loading control and relative quantification compared to cells not transformed with WRB is shown in the graph. Data were calculated from four independent experiments. Error bars indicate standard error. *: p<0.0001; ns: not significant. (TIF) [file pone.0085033.s004.tif]

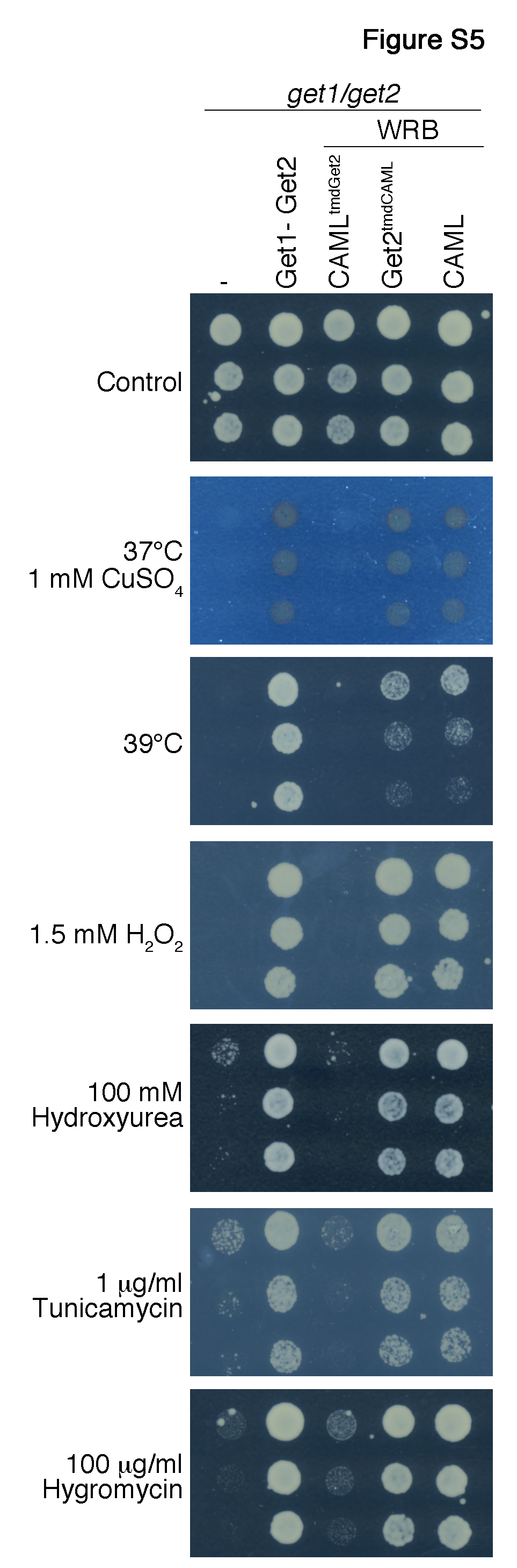

Supplement: Figure S5 — The transmembrane domains of CAML are required to rescue the growth phenotypes of get1/get2 yeast cells. get1/get2 yeast cells were transformed with Get1 and Get2 encoding constructs or WRB in combination with CAML or CAML-Get2 chimeric constructs and serial dilutions spotted on different conditions: HC plates incubated at 30°C (control), 37°C+CuSO4, 39°C, H2O2, hydroxyurea, tunicamycin, hygromycin. (TIF) [file pone.0085033.s005.tif]

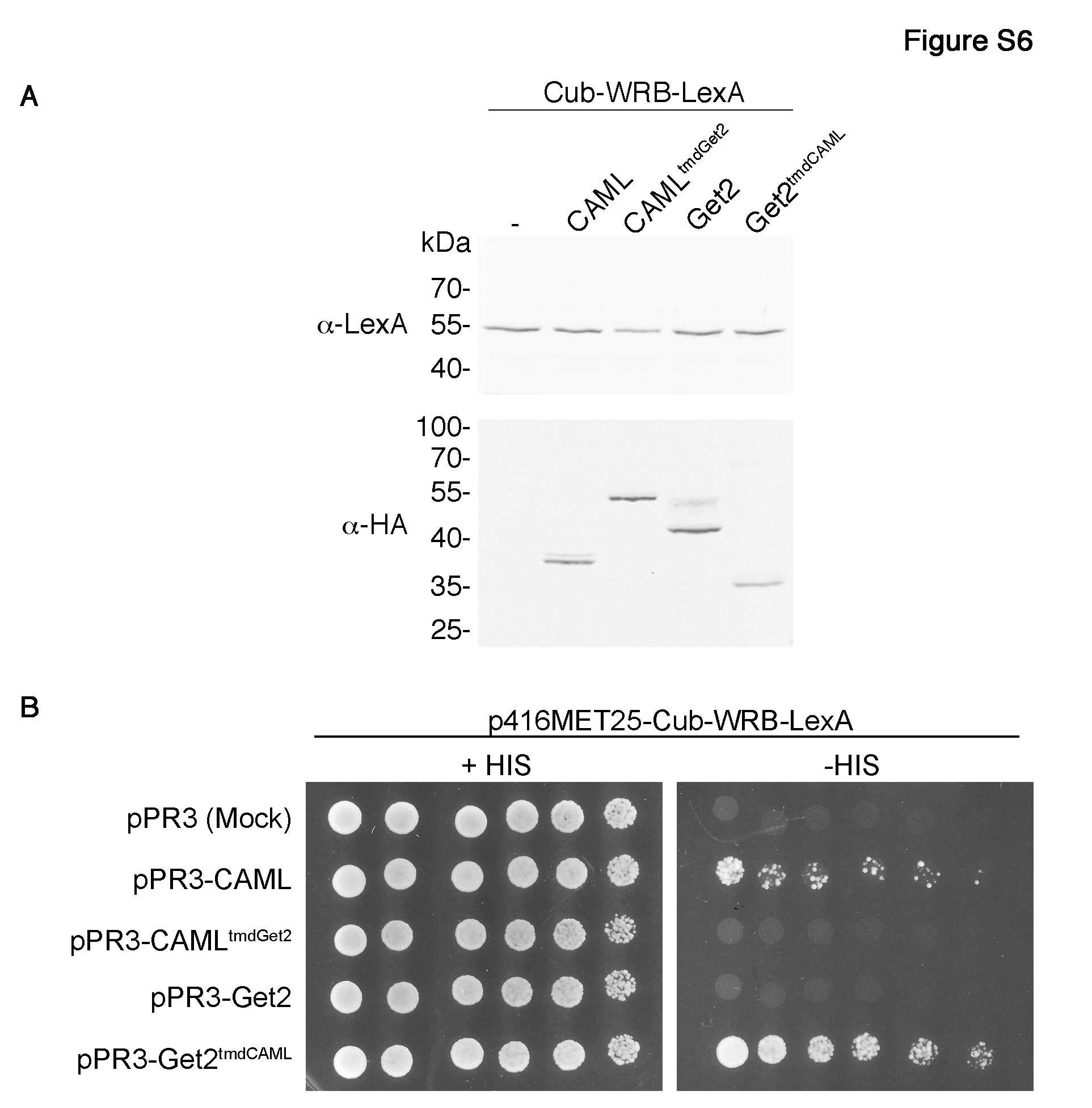

Supplement: Figure S6 — WRB and CAML interact in yeast cells via their transmembrane segments. (A) Yeast cells (NMY51 strain) were transformed with p416-Cub-WRB-LexA in combination with pPR3 (mock), pPR3-CAML, pPR3-CAMLtmdGet2, pPR3-Get2 or pRP3-Get2tmdCAML for split-ubiquitin yeast two-hybrid analysis. Serial dilutions were spotted on HC-ura-trp or HC-ura-trp-his. (B) Protein lysates from NMY51 yeast cells used in split-ubiquitin yeast two-hybrid were separated by SDS-PAGE and analyzed by immunoblot with anti-LexA and anti-HA antibodies. (TIF) [file pone.0085033.s006.tif]

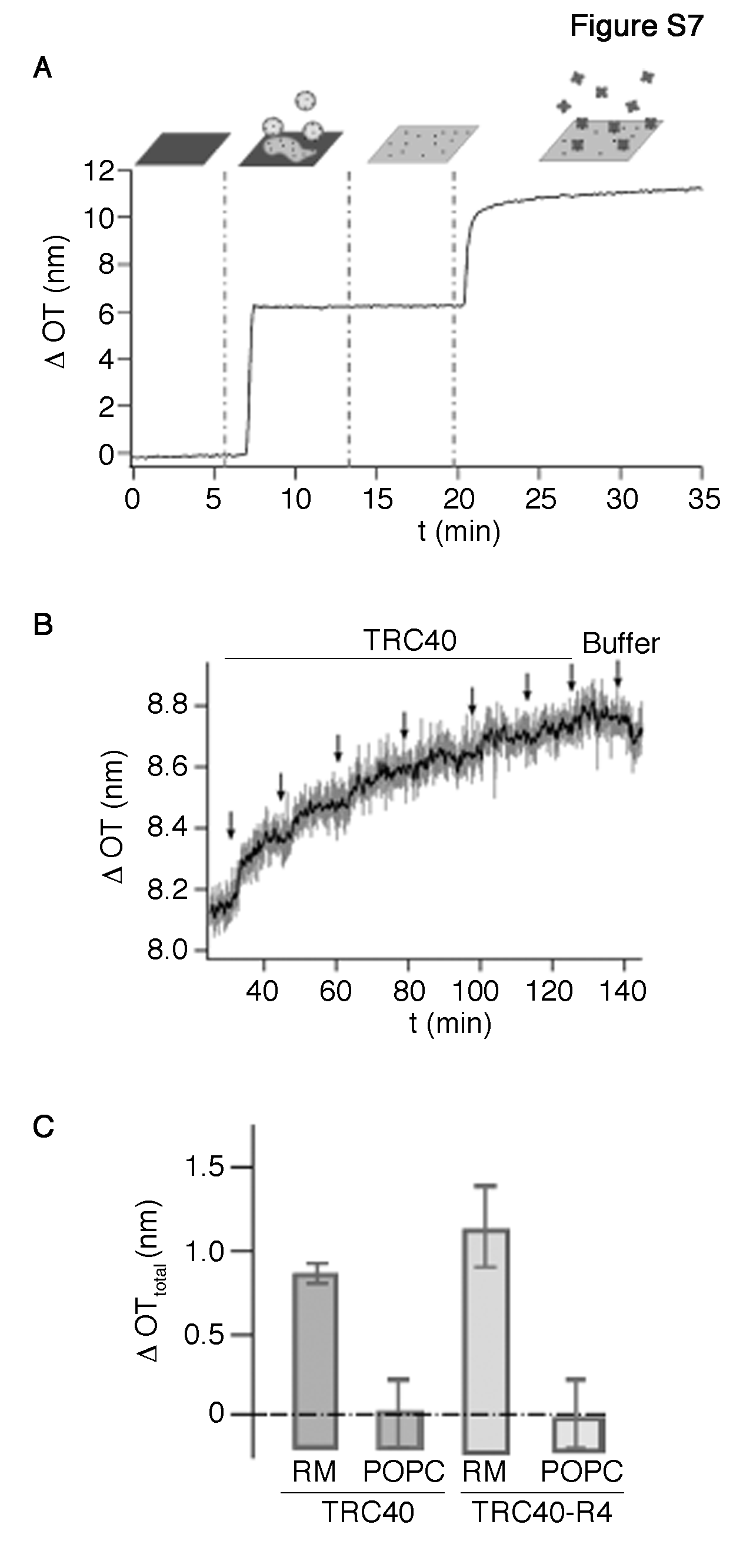

Supplement: Figure S7 — Analysis of TRC40 binding to its membrane receptor complex by Reflectometric Interference Spectroscopy (RIfS). (A) Schematic representation of a RIfS measurement. A mixture of canine pancreatic rough microsomes and POPC liposomes is spread over a silicon-SiO2 chip. Analytes (TRC40 or TRC40-R4) are then injected. Increase in optical thickness (OT) is followed over time. (B) Representative RIfS measurement. Arrows indicate injection of increasing concentration of TRC40 (from 40 nm to 1 µM) and a final buffer washing step. The curve shows variations in OT over time. (C) Final increase in OT at the end of RIfS measurements for experiment performed in presence of microsomes (RM) or pure lipid layers (POPC). (TIF) [file pone.0085033.s007.tif]

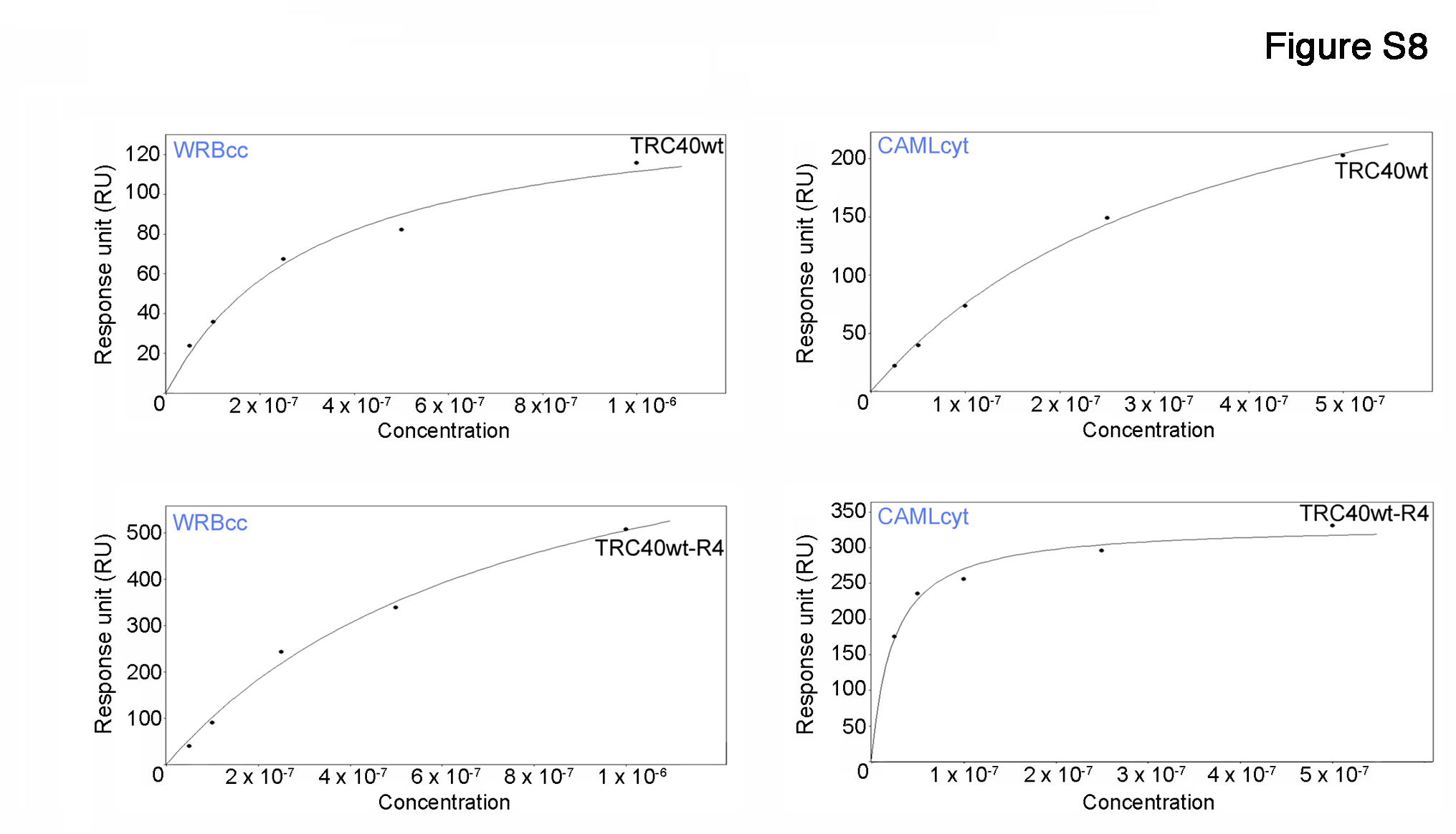

Supplement: Figure S8 — Kinetic characterization of WRBcc and CAMLcyt binding to TRC40. Binding isotherms for TRC40 binding to WRBcc or CAMLcyt calculated by Surface Plasmon Resonance. (TIF) [file pone.0085033.s008.tif]
